# Nutraceuticals: using food to enhance brain health by modulating postnatal neurogenesis in animal models and patient populations

**DOI:** 10.1093/stcltm/szaf006

**Published:** 2025-05-19

**Authors:** Jun Ong, Qingqing Wu, Kazunori Sasaki, Hiroko Isoda, Francis G Szele

**Affiliations:** Department of Physiology, Anatomy and Genetics, University of Oxford, Oxford OX1 3QX, United Kingdom; Department of Physiology, Anatomy and Genetics, University of Oxford, Oxford OX1 3QX, United Kingdom; Alliance for Research on the Mediterranean and North Africa (ARENA), University of Tsukuba, Tsukuba, Ibaraki 305-8572, Japan; Alliance for Research on the Mediterranean and North Africa (ARENA), University of Tsukuba, Tsukuba, Ibaraki 305-8572, Japan; Open Innovation Laboratory for Food and Medicinal Resource Engineering, National Institute of Advanced Industrial Science and Technology (AIST) and University of Tsukuba, Tsukuba, Ibaraki 305-8572, Japan; Faculty of Pure and Applied Sciences, University of Tsukuba, Tsukuba, Ibaraki 305-8571, Japan; Alliance for Research on the Mediterranean and North Africa (ARENA), University of Tsukuba, Tsukuba, Ibaraki 305-8572, Japan; Open Innovation Laboratory for Food and Medicinal Resource Engineering, National Institute of Advanced Industrial Science and Technology (AIST) and University of Tsukuba, Tsukuba, Ibaraki 305-8572, Japan; Faculty of Life and Environmental Sciences, University of Tsukuba, 1-1-1 Tennodai, Tsukuba, Ibaraki 305-8572, Japan; Department of Physiology, Anatomy and Genetics, University of Oxford, Oxford OX1 3QX, United Kingdom

**Keywords:** adult neurogenesis, regenerative medicine, nutraceuticals, pharmacotherapy, hippocampus

## Abstract

Adult hippocampal neurogenesis, while occurring throughout life, decreases with age and in some neurodegenerative diseases. As decreased hippocampal neurogenesis is correlated with cognitive decline, efforts have been made to increase levels of neurogenesis, either through natural compounds, environmental interventions or novel pharmacological compounds. Nutraceuticals are food products with medical benefits such as antioxidation, anti-inflammation or neuroprotection. There has been increasing interest in these “functional foods” and their active compounds in recent years, providing natural alternatives to de novo pharmaceuticals. This review highlights key nutraceuticals that promote neurogenesis and/or improve cognitive outcomes. By outlining the effects of these compounds in the animal models employed and in clinical populations, we also suggest further investigations. We examine common targets and pathways through which these nutraceuticals are believed to exert pro-neurogenic effects. Most nutraceutical preparations contain multiple components, any of which may exert effects on neurogenesis. Identifying key active compounds in nutraceuticals may enable researchers to better understand their effects and standardize doses across studies. The less stringent regulatory requirements for nutraceuticals can be a double-edged sword. While allowing easier access to the beneficial effects, higher doses of these compounds may have detrimental effects. Hence, research in this field should not only aim to identify the benefits of these compounds but also to identify efficacious and safe dosages for them. Our aims are to provide understanding of nutraceuticals, provide evidence for their benefits on neurogenesis and neurogenesis-related behaviors and finally to summarize potential mechanisms and help guide future work.

## Graphical Abstract

**Graphical Abstract:**
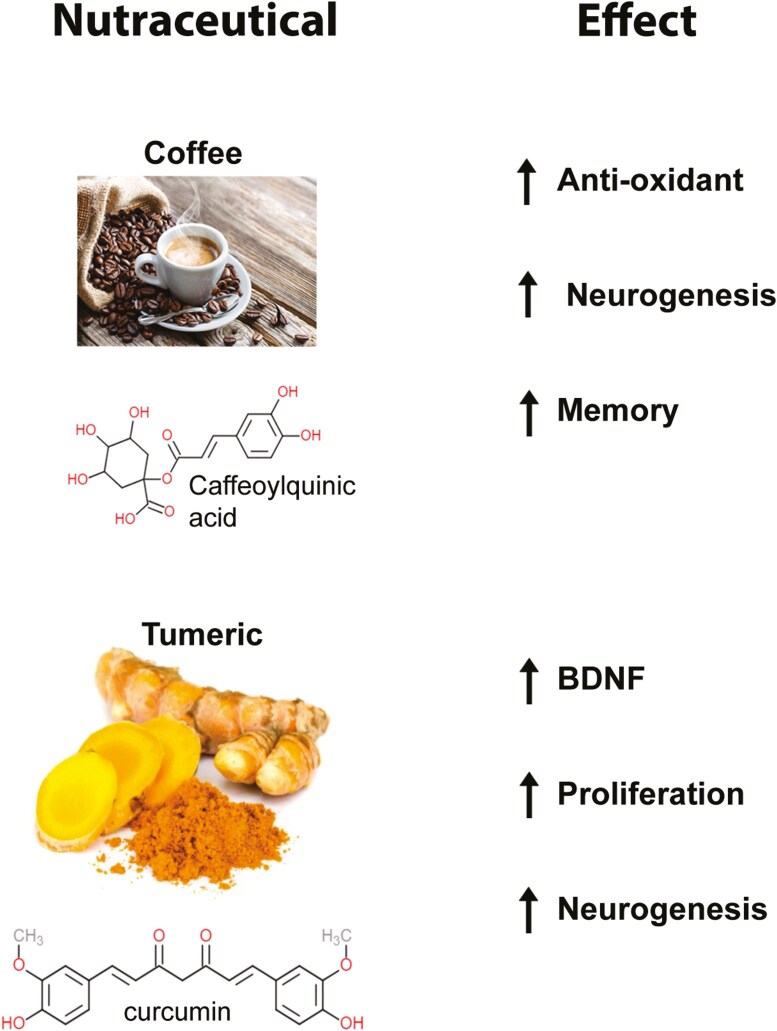

**Significance statement**

Pharmacological treatments for brain disease are in a dismal state and new approaches are needed. Nutraceuticals provide a relatively simple and affordable approach to help cure or treat neurological and psychiatric disorders. We argue that with systematic evaluation of inherent mechanisms and deep toxicology, nutraceuticals can provide a useful alternative.

## Introduction

The prevailing dogma that neurons cannot be generated postnatally was overturned more than 50 years ago by the discovery of adult neurogenesis in the hippocampus and lining the lateral ventricles of rats by Altman and Das.^1^ Over 25 years ago, adult human neurogenesis was definitively demonstrated in patients with cancer.^2^ Recently, the relative levels of human adult neurogenesis has become controversial, however, the majority of histological and transcriptomics studies suggest decreasing but detectable neurogenesis throughout life.^3-8^

The 2 main neurogenic niches are the forebrain subventricular zone (SVZ)^9^ and the hippocampal subgranular zone (SGZ).^10^ A third neurogenic niche is in the hypothalamus, although the rates of cell generation are much lower than in the SVZ and SGZ.^11^ The cells in these niches provide a specialized microenvironment that supports neural stem cell (NSC) activity while harboring neuroblasts with specific properties distinct from that of mature neurons.^12,13^ The niches receive internal and environmental signals that support the integration of adult-born neurons into the mature circuitry.^14^ The discovery of NSCs in the adult brain has granted renewed hope in the plasticity of the mature brain and as a therapeutic source of neurons for neurodegeneration.

### Physiological functions of adult neurogenesis

Extensive research has determined that neurons born throughout life not only replace developmentally born neurons but also perform distinct roles in their newborn and immature states.^13^ SVZ-generated neurons migrate to the olfactory bulbs and have been associated with olfactory learning and memory.^15,16^ While understanding adult hippocampal neurogenesis (AHN) function is incomplete, much evidence points to memory formation. The dorsal hippocampus in mouse encodes spatial memories whereas the ventral hippocampus mediates stressful memories.^17^ Newborn hippocampal neurons can distinguish subtle differences in spatio-temporal patterns of information, termed pattern recognition.^18^ Similar experiences are encoded by distinct patterns of neuronal activity, allowing mammals to distinguish similar events. Evidence from 2-photon calcium imaging in behaving mice demonstrated that immature hippocampal neurons fire at a distinct rate from mature neurons, and are directly involved in context discrimination as confirmed by specific optogenetic silencing.^18,19^

### Injury-induced functions of adult neurogenesis

Brain injury induces adult neurogenesis which can efficiently repair damage in nonmammalian species such as fish and reptiles.^20^ In mammals, adult stem cells attempt injury repair often exemplified by increased proliferation.^21,22^ Injury can also induce SVZ precursor cells to emigrate toward the damage.^21,22^ The emigrated cells provide neuroprotection by reducing inflammation and thus ameliorate functional outcomes. SGZ cells in contrast, seldom migrate far distances after injury but remain in the dentate gyrus of the hippocampus and participate more specifically in improving memory, a function frequently lost in neurodegenerative and neuropsychiatric disorders.^23^

A wide range of brain disorders change basal levels of adult neurogenesis, providing further evidence for their importance. Alzheimer’s disease (AD) is of particular interest since it frequently starts in the hippocampus, the number of newborn neurons decreases in the dentate gyrus and is correlated with cognitive impairment.^5,6^ Furthermore, altered pattern recognition and spatial memory are associated with AD.^24,25^ An excellent review points out AHN involvement in converging mechanisms affecting AD and also major depressive disorder (MDD).^26^ In general, late phases of human AD are associated with decreased neurogenesis,^5,6,27,28^ whereas earlier stages are associated with no change^29^ or increased neurogenesis.^30^ In aggregate, the studies suggest declining neurogenesis in AD contributes to memory loss in late disease stages. AHN has been extensively investigated in AD animal models. In general, single human mutations associated with AD reduce neurogenesis in mouse models.^31,32^ However these are models of rare familial forms of the disease. Some AD mouse models exhibit reduced SVZ proliferation and neurogenesis^33-35^ but increased or unchanged neurogenesis in others.^36,37^ This is similar to the SGZ and likely reflects a complicated combination of model and disease stage differences.

Other diseases such as hypoxia ischemia (H/I) and stroke are more relevant for the SVZ since they induce neuroblast emigration to the injury. H/I reduces blood flow and oxygen concentrations causing new-born brain injury and affecting 0.3% of all births annually.^38^ The Rice-Vannucci animal model faithfully recapitulates many aspects of H/I induced disease including cell death in the forebrain.^39,40^ Rodent H/I activates NSCs and neuroblasts in the postnatal SVZ and they emigrate toward the injury.^22,41^ Similarly, stroke activates human SVZ proliferation and SVZ cell migration to the injury.^42-44^ Neurogenesis inhibition with genetic approaches worsened symptoms caused by rodent stroke.^45,46^ Supporting this, stimulating neurogenesis in animals ameliorates H/I symptoms.^22^ Thus, adult neurogenesis is clearly dynamic, and the field is now at a transition phase in which molecular targets to increase neurogenesis are being sought.

### Known modulators of adult neurogenesis

A wide range of internal and external molecular and environmental cues affect adult neurogenesis. Neuronal network activity, neurotrasmitters, neurotrophic and growth factors, signaling molecules and transcription factors regulate neurogenesis.^47-56^ Additionally, environmental stimulation, exercise, learning, and social interaction increase neurogenesis while aging and stress diminish new neuron production.^57-62^ Interestingly, newborn neuron numbers can be controlled at multiple lineage stages yet most studies do not distinguish between production versus survival. Notably, pharmacological intervention such as with selective serotonin reuptake inhibitors (SSRIs) can increase neurogenesis.^63^ In this review we argue that nutraceuticals, dietary supplements and related substances such as medicinal plants could be administered to augment neurogenesis in health and disease.

Several studies have indicated that diet influences stem cells and adult neurogenesis (Table 1).^64,65^ Caloric restriction increases overall organismal longevity and specifically newborn neuron survival in the DG.^66^ Conversely, high-glucose/high-fat diets have been associated with decreased AHN, along with disrupted cognitive function.^67,68^ However, caloric restriction mimetics including resveratrol and metformin enhance spatial memory, neuroprotection of primary cortical neurons in vitro and mitochondrial function in mouse models of AD.^69,70^ Nevertheless, a causal link between the mimetics and dietary restriction with adult neurogenesis still needs to be established. As well, several limitations constrain the use of dietary restriction or glucose intervention in promoting neurogenesis and cognitive health. Besides potential toxicity, dietary restrictions are not sustainable as they rely on compliance and lifestyle restrictions. Nevertheless, the poor health consequences of obesity and high body mass index, and the positive effects of low caloric diet on neurogenesis together make a strong case for decreasing caloric intake in the majority of the population.

**Table 1.** Examples of nutraceuticals with a summary of their effects on neurogenesis in various models.

Nutraceutical	Category and info	Effects	Proposed mechanisms		Limitations	Ref
3,4,5-triCQA caffeoylquinic acid	Polyphenol Derivative of CQA	**Model: SAMP8 mice** MWM rescue Increased neurogenesis in DG but not SVZ **Model: hNSCs cells** Decreased ROS in differentiated cells Induced differentiation and differentiation-related gene expression Increased neuronal development and axonal transport genes	Gene expression of treated hNSCs suggests changes in cell cycle, chromatin remodeling, neuronal development, actin cytoskeleton and neurite extension		Proposed mechanisms may be effect not cause	^81^
Verbenalin	Iridoid glucoside Found in medicinal herbs of Verbenaceae family	**Model: human amnion epithelial cells (hAEC)** spheroids Microarray, RT-PCR validation **Model: SH-SY5Y cells** MTT showed protection against Aβ-induced cytotoxicity Ameliorated Aβ -induced ATP decline and Aβ-induced ROS levels Regulated gene expression of EGFR, VEGF and NRG1 in Aβ -induced cells	Microarray suggests changes in neurogenesis, neuron differentiation, nervous system development, neuromuscular process, AD-related genes RT-PCR validated ErbB signaling (AD-related), Rho family genes and circadian entrainment genes		Results do not directly show increases in neurogenesis and could be strengthened with experiments showing rescue of neurogenesis and gene expression changes in disease models While hAECs are potentially less tumorigenic than iPSCs or ESCs, they are also more heterogenous than other stem cell sources.	^130^
Ferulic Acid	Phenolic phytochemical	**Model: ICR mice** Tail suspension test showed decreased immobility time with FA treatment, similar to antidepressant bupropion. Increased dopamine, noradrenaline and BDNF levels, and increased brain ATP levels	Microarray showed upregulation of energy metabolism, cell survival and proliferation and dopamine synthesis genes		While increased BDNF is associated with increased neurogenesis, no direct measurements of levels of neurogenesis was performed.	^131^
Curcumin	Polyphenol	**Model: C57 mice** Increased neurogenesis in DG (DCX) Increased BDNF levels **Model: C17.2 NPCs** Increased proliferation	Increased NF1X levels regulated increased proliferation in NPCs			^132^
Folic acid	Synthesized from folate by gut bacteria Important in nervous system development	**Model: adult female Sprague-Dawley rats** Folic acid dose-dependently influenced neurogenesis with low levels decreasing but high levels increasing neurogenesis in the ventral hippocampus, suggesting this region, which is important for regulating stress, is particularly sensitive to folic acid in diets. Addition of SST to prevent folic acid synthesis negated the effects of 5-MTHF to increase neurogenesis in the ventral hippocampus.			Discussed in the context of depression rather than cognitive impairment No behavioral tests for hippocampal dependent tasks	^113^
SBL88	Heat-killed dietary lactobacillus Brevis	Model: C57bl/n male mice Improvement of hippocampus-dependent social recognition and contextual fear memory Enhanced adult hippocampal neurogenesis (survival but not proliferation)				^120^
Ethanol extract of Aurantiochytrium sp. (EEA)	Microalgae extract *Aurantiochytrium* sp. Strain 18W-13a accumulates squalene	Model: ICR mice EEA reduced immobility time during forced swim test similar to antidepressant imipramine EEA induced expression changes in genes associated with inflammation, dopaminergic, cholinergic, glutamatergic, and serotonergic synapses. EEA decreased gene expression of proinflammatory cytokines TNFα and IL-6, while increasing BDNF Model: SHY-5Y cells Improved cell viability, reduced dexamethasone-induced vell death	Regulation of pro-inflammatory genes contribute to changes in neurotransmitters, resulting in reduced neuroinflammation and neurotransmitter modulation that lead to antidepressant effects		Behavioral assays were depression and not dementia model No direct investigation of changes to neurogenesis in mice	^121^
l-Theanine	γ-glutamylethylamide amino acid ingredient in green tea structural analogue to l-glutamine (not l-glutamic acid)	**Model: cultured neurons and astroglia** Marked inhibition of [3H]L-GLN uptake without affecting [3H]L-GLU uptake **Model: mouse primary neural progenitor cells (neonatal)** Sustained exposure to l-theanine lead to upregulation of L-GLN transporter isoform Slc38a1 Promotion of both proliferation and neuronal commitment are seen along with marked acceleration of the phosphorylation of mammalian target of rapamycin (mTOR) and relevant downstream proteins. **Model: p19 cells (pluripotent embryonic carcinoma)** Stable overexpression of Slc38a1 leads to promotion of cellular growth with facilitated neuronal commitment marked phosphorylation of mTOR and downstream proteins in a fashion insensitive to additional stimulation by l-theanine **Model: mouse primary neural progenitor cells (adult)** Increase of neurosphere size with l-theanine treatment	Upregulation of Slc38a1 by l-theanine could activate mTOR signalling, promoting cell growth and hence neurogenesis	Requirement of sustained exposure— might not be feasible as a pharmacological target		

## Nutraceuticals modulate neurogenesis and promote cognitive health

**Figure 1.**
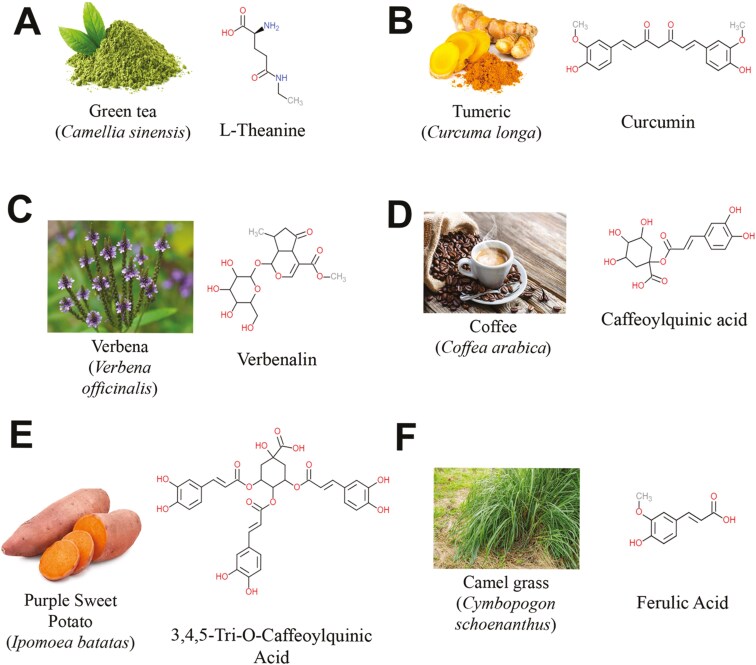
Examples of the sources of nutraceuticals and the chemical structures found within them and the bioactive chemical structures.

Specific dietary effects on adult neurogenesis and cognitive health have been explored (Table 1). Potential neurogenic and neuroprotective effects associated with anti-oxidation and metabolic function^71^ have propelled research into dietary compounds that might influence symptoms of neurodegeneration, paving the way for the nutraceuticals field. Given findings of the dysregulation of neurogenesis in many CNS disorders, a number of recent studies have examined the potential of nutraceuticals as therapeutic candidates for the disorders described above by promoting adult neurogenesis (Figure 1).

Foods that promote health have been used in medicine for millennia. The modern term “nutraceutical” can be traced to a review by DeFelice in 1995: “any substance that is a food or part of a food and provides medical or health benefits, including the prevention and treatment of disease.”^72^ Indeed, the nutraceutical field had its beginnings as early as the 1980s in the United States, with initial studies examining potential health benefits of fish oil. The subsequent surge in scientific studies has covered a wide range of potential nutraceuticals, from β-carotene for cancer to garlic for atherosclerosis. This paved the way for scientific attention and validation of nutraceuticals with potential clinical benefits.

Nutraceuticals are a fast growing industry providing a large variety and in some cases vast sources of alternative therapeutic agents.^73^ Nutraceuticals could be particularly advantageous and attractive to industry as they typically have better patient compliance compared to drugs, have fewer safety concerns, require fewer regulatory hurdles and need significantly less financial investment to reach the market. A major limitation however is that the molecular constituents of foodstuffs, plants and traditional medicines can vary greatly across sources and over time. Compounding this problem, there is relatively little research breaking down which constituent or combination of constituents provide the benefits. Thus reproducibility and confirmation of efficacy across laboratories remains a challenge.

There are 3 main, overlapping, categories of nutraceuticals, namely functional foods, dietary supplements, and herbal/natural products.^72^ Their cellular effects, can include multiple functions: melatonin, for instance is a known antioxidant, yet also promotes neurogenesis and affects metabolism.^74^

### Flavonoids

Flavonoids are naturally occurring phenolic phytochemicals many of which have medicinal benefits including anti-inflammatory and anti-oxidative effects. Some of these have been shown to be neuroprotective and promote neurogenesis.^75,76^

### Caffeoylquinic acid and 3,4,5-tricaffeoylquinic acid

Caffeoylquinic acid (CQA) is a phenylpropanoid abundant in coffee beans, sweet potatoes, and propolis (resinous adhesive substance made by bees for hive maintenance). Studies have associated CQA and its derivatives with a broad range of potentially beneficial biological effects, including anti-oxidation, hepatoprotection, anti-bacterial, anti-histaminic, and anti-cancer effects.^77,78^ In vitro experiments have shown that this compound exhibits neuroprotective effects^79^ while CQA-rich sweet potato extract given to the SAMP8 mouse model of aging and sporadic AD induces protein expression related to antioxidant activity, energy metabolism, and neuronal plasticity pathways in the CNS.^77^ These data suggest CQA benefits cognitive health, however, direct evidence demonstrating the effects of CQA on memory function, as well as on adult neurogenesis, are still lacking.

**Figure 2.**
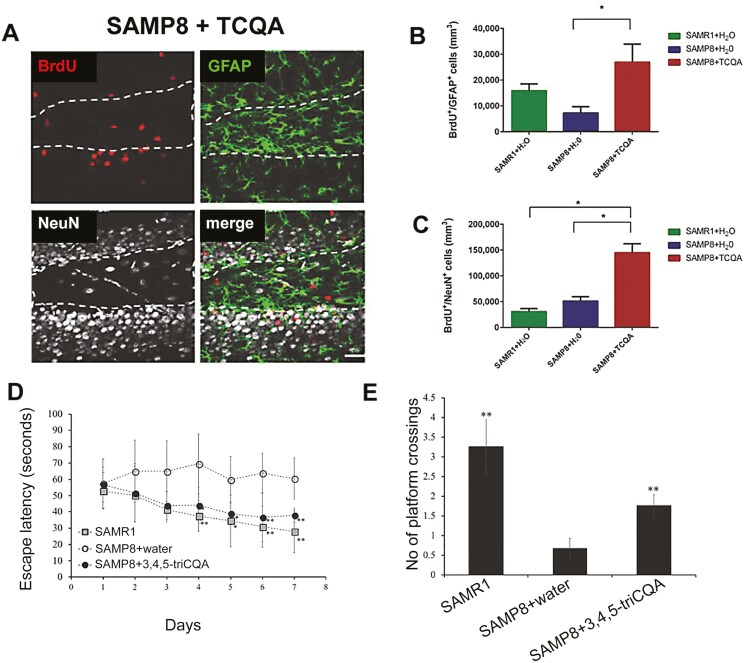
TCQA increases neurogenesis and ameliorates spatial memory**. (**A–C) Effect of oral administration of 3,4,5‐triCaffeoylquinic acid (TCQA) on hippocampal stem cell activation and neurogenesis. SAMP8 mice were administrated with TCQA (5 mg/kg) for 30 days. (A) Photomicrographs show coronal sections containing the dentate gyrus processed for immunohistochemical detection of proliferating BrdU+ cells and GFAP, a protein expressed by stem cells in the DG. NeuN is a mature neuronal marker. (B) Graph represents the number of BrdU+ cells that co‐express GFAP. (C) Graph represents the number of BrdU+ cells that co‐express NeuN. Thus TCQA increases the number of hippocampal stem cells and newborn neurons. (D) TCQA increased spatial learning and memory as determined by escape latency of senescence‐accelerated resistant mouse 1 (SAMR1) mice, senescence‐accelerated prone mouse 8 (SAMP8) mice and SAMP8 TCQA‐treated group determined by Morris water maze (E) TCQA increased the numbers of platform crossings in the SAMP8 treated compared to SAMP8 water treated mice. (Adapted with permission from 81).

A CQA derivative, 3,4,5-triCQA (TCQA) is a natural compound proposed for the treatment and prevention of age-related diseases. Among CQA derivatives, TCQA induces the highest increase in ATP in vitro.^80^ Adult SAMP8 mice given TCQA for 30 days showed spatial memory deficit reversal in the Morris water maze (MWM), demonstrating its potential to boost cognitive health.^81^ TCQA also increased hippocampal neurogenesis as evidenced by increased numbers of BrdU^+^GFAP^+^ stem cells and BrdU^+^NeuN^+^ newborn neurons. In the SVZ, the number of BrdU^+^ cells increased upon TCQA administration (Figure 2). There was no increase in the number of SVZ BrdU^+^GFAP^+^ stem cells suggesting TCQA activates the niches differently. This may be beneficial as certain diseases may require stem cell activation in one niche but not the other. For example, if the disease target is only memory loss, SGZ activation-only would be required. TCQA increased cell viability, differentiation, cell cycle progression, metabolic function and decreased ROS levels in vitro in human NSCs suggesting it uses multiple mechanisms.^81^ Analysis of gene expression changes on TCQA treatment using microarray show upregulation of DYRK2 and RIF1, suggesting it suppresses G1/S transition and promotes G0/G1 arrest. This suggests that TCQA could regulate proliferaton of neural stem cells.^81^

### Curcumin

Curcumin, a phenol and major component of the turmeric plant (*Curcuma longa*), has been traditionally used in India to treat oxidative stress or inflammation related diseases.^66^ While modern research into curcumin has predominantly focused on cancer prevention, a number of reports have associated curcumin treatment with the stimulation of embryonic NSCs and adult neurogenesis in the hippocampus.^82-84^ Furthermore, based on epidemiologic data, regular curcumin intake from curry has been associated with better cognitive function in healthy elderly humans.^85^ Clinical trials of curcumin for treatment of Alzheimer’s Disease,^86^ ulcerative colitis,^87^ and post-operative inflammation^88^ have demonstrated promise in ameliorating inflammatory-linked diseases besides cancer.

Curcumin enhances memory function and/or reverses cognitive impairment in rodents.^89-91^ For example, feeding aged rats curcumin for 6 weeks enhanced social recognition memory while curcumin feeding for 12 weeks enhanced MWM spatial memory, concomitant with an increase in hippocampal neural proliferation.^92^ Curcumin also induced significant changes in expression of genes involved in neurotransmission, metabolic homeostasis and neuronal development.^92^ However the authors did not investigate validated targets from their gene expression screen. Gene expression profiles were also drastically distinct between 6- and 12-week treated animals, suggesting significant variability. Nonetheless the results highlighted the potential influence of curcumin on the brain, impacting gene expression of significant processes including neuronal development and neurotransmission while guiding future exploration into these molecular targets.^92^ Another study showed that curcumin reversed stress-related AHN deficits in rats. Concomitantly, they revealed that the serotonin receptor 1A and brain-derived neurotrophic factor (BDNF) affected curcumin-induced modulation of stress.^91^ Kim et al. corroborated curcumins’ effect on AHN in mice, and implicated ERKs and p38 kinases signal transduction pathways, which regulate neuronal plasticity and stress.^84^

Exon array analysis of cortical and hippocampal mRNA transcription revealed that curcumin induces modulation of genes involved in neurotransmission, neuronal development, signal transduction, and metabolism.^93^ Additionally, curcumin affects oxidative stress activity,^94^ activates Nrf2-dependent antioxidant^95-97^; BDNF levels, ERK/P38 kinase signaling,^98^ PKCδ degradation^99^ and/or histone acetyltransferase inhibition.^100^ Moreover, curcumin has a potential neuroprotective effect, having been shown via in vitro and in vivo studies to reduce amyloid-beta build-up.^101^ Low concentrations of curcumin lead to mitogenic action mediated by ERK and p38MAPK.^84^ High doses of curcumin which are used to trigger apoptosis in cancer cells, also increase oxidative stress which has been shown to negatively modulate adult neurogenesis.^102^ These findings are exciting but tempered by the potential dose-dependent toxicity of curcumin.^103^

### Capsaicin

Capsaicin (trans-8-methyl-N-vanillyl-6-nonenamide), from hot red peppers, diminishes AHN; potentially by blocking ERK signaling.^104^ We do not know if this affects memory but suggests the involvement of ERK in AHN. Capsaicin highlights the importance of understanding the effects of diet on neurogenesis and potentially on cognition, to maximize their benefits and minimize harm.

### Amino acids

Theanine, an amino acid with a molecular structure similar to glutamate, is present in green tea such as Gyokuro and Matcha.^105^ Theanine inhibits [^3^H]Glutamine uptake without affecting [^3^H]Glutamate uptake in rat brain synaptosomes, analogous to glutamic acid.^105,106^ In vitro theanine exposure promotes growth^107^ while in a mouse model of chronic stress, theanine alleviates learning impairment, in addition to mitigating shortened lifespan, cerebral atrophy, behavioral depression and oxidative DNA damage.^108^

Theanine’s brain mechanisms are unclear, but theanine exposure in neurospheres increased mTOR pathway protein phosphorylation, neurosphere numbers, BrdU incorporation and expression of the neuronal marker MAP2 ^107^. However, an AD cell model showed theanine can induce neurotoxicity, by acting as an antagonist at N-methyl-D-aspartate (NMDA) receptors.^109^ These findings were mainly based on in vitro studies, and validation is required in vivo to clarify the effects of theanine.

### Folate

Folate, the natural form of vitamin B9 present in food, is an essential macronutrient. Mammals cannot synthesize folate, so supplementation is required, as for its synthetic forms: folic acid, folinic acid and 5-methyltetrahydrofolate (5-MTHF).^110^ Folate is well-known for being vital during embryonic and childhood development; folate deficiency causes neural tube defects, low infant birthweight, increased risk of miscarriage and other complications.^111,112^

Recent evidence suggests folate might also be critical for neurogenesis in adulthood. In adult rats, folic acid increased hippocampal neurogenesis in a dose-dependent manner.^113^ Separately, in a rat model of cerebral ischemia, folic acid reversed ischemia-related cognitive impairment on the Y-maze.^114^ Moreover, hippocampal neurogenesis was robustly increased, indicated by cells double-positive for BrdU and the neuronal marker NeuN (BrdU^+^NeuN^+^). Notch1, Hes1 and Hes5, components of the Notch signaling pathway, were increased after ischemia, and further stimulated with folic acid supplementation.^114^ Experimental stroke via middle cerebral artery occlusion (MCAO) might lead to increased Notch signaling as a compensatory mechanism, and folic acid supplementation would thereby further stimulate Notch increasing this regenerative effect.

### Heat-killed bacteria

Gut-brain biota have received much attention since the landmark finding of their necessity in early life for stress responsiveness in adulthood.^115^ Indeed, *Lactobacillus* microbiota has several profound benefits on health, and is used to enhance the immune system and intestinal function.^116-119^*Lactobacillus brevis* (SBC8803) enhances hippocampus-dependent social recognition memory and contextual fear memory.^120^ This was associated with elevated numbers of progenitor cells in the SGZ due to increased survival instead of proliferation.^120^ Further work is required to elucidate the effects of heat-killed bacteria on neurogenesis, elucidate their comparative potencies and underlying molecular changes.

### Algae

Algae, are a valuable food source with health benefits and are readily available since they are harnessed as a fuel source. The novel 18W-13a strain of *Aurantiochytrium* sp., an oleaginous microalgae in the *Thraustochytriaceae* family, reduced depressive symptoms in mice as measured by the forced swim test.^121^ Indeed, its anti-depressive-like effects were comparable to the antidepressant, imipramine.^121^ Ethanol extract of *Aurantiochytrium* (EEA) increased AHN as assessed by BrdU+GFAP+ and BrdU+NeuN+ cells, suggesting that both the number of stem cells and the number of newborn neurons was increased, respectively.^121^ EEA-treated mice had significantly altered expression of genes related to inflammation as well as dopaminergic, cholinergic, glutamatergic, and serotonergic neurotransmission.^121^ Given the strong association between neuroinflammation and neurogenesis, it was not surprising that this microalgae strain increased learning and memory. Indeed, future work is warranted to explore, potential benefits of other microalgae strains.

## Discussion

There is a vast and growing body of literature supporting myriad CNS benefits of nutraceuticals, particularly in terms of physiological and cognitive health. In this review we summarized selected key nutraceuticals found to modulate learning and memory concomitant with adult neurogenesis. Moving beyond the behavioral effects of nutraceuticals, the field is at a turning point where a number of studies have pointed to a wealth of mechanistic targets that nutraceuticals may modulate, and thereby exert effects on cognitive health. While evidence is largely correlative for now, these studies shed light on the future direction of nutraceutical research in delineating how nutraceuticals cause brain health.

### BDNF signaling

BDNF signaling is both a prominent target of nutraceuticals and a stimulator of AHN and memory function.^91,122,123^ Oral supplementation of curcumin to chronically stressed rats elevated BDNF and serotonin receptor 1A expression, for instance, while increasing neurogenesis in the SGZ.^98^ Capsaicin, another flavonoid, was found to inhibit neurogenesis while suppressing ERK signaling and BDNF expression.^104,124^ Given that our understanding is still based on limited, and largely in vitro, evidence, further investigations are warranted to delineate the precise role of BDNF in nutraceutical effects on memory and neurogenesis. The potential role of BDNF suggests neurotrophin mediation may also be a mechanism of nutraceutical-induced adult neurogenesis and cognitive health. For example neurotrophin-3 (NT-3) is necessary for adult NPC differentiation in vivo and may be a causative factor in AHN and spatial memory.^125^ Future neurotrophin studies in AHN might reveal similar interesting mechanisms.

### TCQA signaling

Another flavonoid, TCQA, enhances spatial memory while promoting neurogenesis in SAMP8 mice.^81^ Microarray data from the same study provided a number of plausible molecular targets. For instance, TCQA increased expression of bone morphogenetic protein (BMP) signaling genes including the *BMP5* ligand and BMP receptor type II (*BMPR2*), downstream SMAD family member 5 (*SMAD5*) and neuronal differentiation 1 (*NEUROD1*), factors necessary for cellular differentiation and proliferation. The study on folate by Zhang et al. suggested that Notch signaling may be involved, as protein levels of Notch1, Hes1, and Hes5 were further increased in a rat model of ischemia treated with folic acid supplementation,^114^ while amino acid theanine treatment elevated mTOR signaling proteins, purportedly involved in neural proliferation and adult neurogenesis.^107^

### Curcumin signaling

Genes involved in neurotransmission, neuronal development, signal transduction, and metabolism were revealed by exon array analysis of cortical and hippocampal mRNA after curcumin.^92^ Curcumin also has positive effects on oxidative stress^94^ and activates the Nrf2 antioxidant response pathway.^95-97^ It is likely that most nutraceuticals will stimulate multiple signaling pathways and gene regulatory networks. Besides the signaling molecules already mentioned, curcumin also modulates ERK/P38 kinase signalling,^98^ PKCδ degradation,^99^ histone acetyltransferase inhibition,^100^ and reduce amyloid-beta build-up,^101^ each providing critical targets for future work.

### Gender differences

Males and females can respond variably to nutraceuticals and disease. There is increasing evidence for sex differences in the action of anti-inflammatory drugs, with women being at significantly higher risk of adverse effects.^126^ The anti-thrombotic activity of selective and non-selective COX-inhibitors tends to be stronger in men than women.^126^ Differences in neurogenesis between the brains of males and females have been demonstrated,^127,128^ suggesting differentially regulated pathways for cognitive variations and susceptibility to diseases. Finally, females are under-represented in clinical trials^129^ despite the fact that women have distinct physiology and signaling.

### Clinical use of nutraceuticals

Many of the nutraceutiucals discussed above have direct implications for clinical use.^101^ For example Curcumin treatment in vitro and in vivo decreased amyloid-beta build-up, a key pathological hallmark of AD.^101^ What is needed to apply these research results to human diseases? It is crucial to harness these benefits effectively and safely. Due to less stringent requirements for nutraceuticals, relative to small molecule drugs entering the market, there is greater potential for incorrect usage. For example, higher doses of curcumin increase cancer risk.^102^ Dose concerns such as this should be carefully evaluated for safety and efficacy. Another critical aspect of nutraceutical use in the clinics, is that individual component compounds should be resolved and tested for efficacy and toxicity in animal and cell models. While a food source might promote cognitive health in patients and even in healthy controls, understanding its molecular components and their individual effects on cognitive health as well as neurogenesis would enable researchers to maximize benefits.

## Conclusions

The current literature has established strong evidence for the cognitive benefits of various nutraceuticals, specifically in enhancing neurogenesis and hippocampal-dependent memory. Given the broad definition of “nutraceuticals,” a large number of food and food compounds fall under this umbrella term, giving rise to a multitude of different effects on cognitive health, neurogenesis, and likely a range of distinct causative mechanisms. This presents the field with a vast array of compounds to investigate, along with the challenge of delineating the effects and distinct mechanisms of each compound separately, synergistically and dose-dependently.

A limitation in the field is that many studies show correlative evidence for underlying mechanisms, yet whether these molecular and cellular targets are directly causative often remain to be proven. Furthermore, most studies focus on SGZ neurogenesis rather than SVZ, potentially because the SGZ is more crucial for nutraceutical-related enhancement of memory and cognitive health. Nevertheless, we predict that nutraceutical-induced augmentation of SVZ neurogenesis will be beneficial in brain injury repair. The SGZ has been extensively researched with established hippocampal-related behavioral tasks. Finally, there is need for studies to evaluate not only neural proliferation as in most papers, but also stem cell quiescence and activation, differentiation, functional integration into the mature circuitry and newborn cell survival, all of which could be regulated by nutraceuticals.

Importantly, direct evidence is still required to prove causation  between nutraceutical supplementation, neurogenesis and cognitive health. Molecular targets, which may be distinct to each nutraceutical, should be further investigated to understand their mechanisms of action; while the effect of active compounds or components should be titrated to maximize efficacy. In this way, the burgeoning field can streamline and maximize the full potential of nutraceuticals, as a relatively untapped vital, natural and non-invasive resource for improving brain health and preventing aging diseases such as mild cognitive impairment and AD.

## Data Availability

No new data were generated or analysed in support of this research.
